# KIF1C and new Huntingtin-interacting protein 1 binding proteins regulate rheumatoid arthritis fibroblast-like synoviocytes’ phenotypes

**DOI:** 10.3389/fimmu.2024.1323410

**Published:** 2024-04-25

**Authors:** Teresina Laragione, Carolyn Harris, Percio S. Gulko

**Affiliations:** Division of Rheumatology, Department of Medicine, Icahn School of Medicine at Mount Sinai, New York, NY, United States

**Keywords:** signaling, fibroblast, rheumatoid, synovitis, synovial, invasion, actin

## Abstract

**Background:**

Huntingtin-interacting protein-1 (HIP1) is a new arthritis severity gene implicated in the regulation of the invasive properties of rheumatoid arthritis (RA) fibroblast-like synoviocytes (FLS). These invasive properties of FLS strongly correlate with radiographic and histology damage in patients with RA and rodent models of arthritis. While HIP1 has several intracellular functions, little is known about its binding proteins, and identifying them has the potential to expand our understanding of its role in cell invasion and other disease-contributing phenotypes, and potentially identify new targets for therapy.

**Methods:**

FLS cell lines from arthritic DA (highly invasive) and from arthritis-protected congenic rats R6 (minimally invasive), which differ in an amino-acid changing HIP1 SNP, were cultured and lysed, and proteins were immunoprecipitated with an anti-HIP1 antibody. Immunoprecipitates were analyzed by mass spectrometry. Differentially detected (bound) proteins were selected for functional experiments using siRNA knockdown in human RA FLS to examine their effect in cell invasiveness, adhesion, cell migration and proliferation, and immunofluorescence microscopy.

**Results:**

Proteins detected included a few known HIP1-binding proteins and several new ones. Forty-five proteins differed in levels detected in the DA versus R6 congenic mass spectrometry analyses. Thirty-two of these proteins were knocked down and studied *in vitro*, with 10 inducing significant changes in RA FLS phenotypes. Specifically, knockdown of five HIP1-binding protein genes (CHMP4BL1, COPE, KIF1C, YWHAG, and YWHAH) significantly decreased FLS invasiveness. Knockdown of KIF1C also reduced RA FLS migration. The binding of four selected proteins to human HIP1 was confirmed. KIF1C colocalized with lamellipodia, and its knockdown prevented RA FLS from developing an elongated morphology with thick linearized actin fibers or forming polarized lamellipodia, all required for cell mobility and invasion. Unlike HIP1, KIF1C knockdown did not affect Rac1 signaling.

**Conclusion:**

We have identified new HIP1-binding proteins and demonstrate that 10 of them regulate key FLS phenotypes. These HIP1-binding proteins have the potential to become new therapeutic targets and help better understand the RA FLS pathogenic behavior. KIF1C knockdown recapitulated the morphologic changes previously seen in the absence of HIP1, but did not affect the same cell signaling pathway, suggesting involvement in the regulation of different processes.

## Introduction

Rheumatoid arthritis (RA) is one of the most common autoimmune diseases affecting nearly 1% of the population and is associated with reduced quality of living and increased risk for disability (1–3). New treatments have been developed over the past two decades (4–7), but disease remission remains uncommon.

Disease severity and joint damage are major predictors of disease outcome and the risk for disability, yet little is known about genes implicated in these processes (8–10). Identifying severity and joint damage genes has the potential to generate novel targets for treatment and prognostication. A promising novel strategy to identifying disease severity genes may involve focusing on the fibroblast-like synoviocytes (FLS) (11–13). The RA FLS has a central role in arthritis pathogenesis and has an altered and invasive phenotype (14). The RA FLS behavior resembles that of cancer cells with increased cell numbers, increased local invasiveness that does not respect tissue boundaries, increased longevity, and increased expression of oncogenes and proteases among others (12, 14–18). Importantly, the *in vitro* invasive properties of the FLS correlate with radiographic joint damage in patients with RA (19) and with histology damage in rodent models (12).

We have recently identified Huntingtin-interacting protein 1 (HIP1) as a new mediator of arthritis severity and joint damage (20). HIP1 mediates receptor tyrosine kinase (RTK) activation via Rac1 to control FLS invasiveness. HIP1 regulates different cellular functions including actin cytoskeletal, clathrin-dependent receptor endocytosis, and is a nuclear receptor chaperone (21). It is also expressed in increased levels in some cancer (22, 23) and capable of transforming cells and increasing accumulation of RTK such as EGFR (24). However, little is known about HIP1-binding proteins and those have the potential to be more tissue specific and less ubiquitous than HIP1, and perhaps better targets for treatments. In the present study, we describe the identification of new HIP1-binding proteins in both rats and human FLS and implicate 10 of them in the regulation of phenotypes relevant to FLS behavior in RA.

## Materials and methods

### Congenic and subcongenic breeding

DA.ACI(Cia25-R6) congenic breeding was previously reported (20, 25). Briefly, we used a genotype-guided strategy to introduce arthritis-resistant ACI alleles at the HIP1-containing interval into DA/HsdNsi (DA), followed by the intercrossing of identical recombinants to generate homozygous subcongenics.

### Isolation and culture of fibroblast-like synoviocytes (FLS)

FLS (human and rodent) were obtained as previously described (20, 26). Briefly, rat synovial tissues were obtained from the ankle joints after euthanasia. The skin was extensively cleaned with ethanol, followed by removal of the skin exposing the joint capsule. The joint capsule was then sectioned, exposing the synovial tissue for excision. Freshly obtained synovial tissues (human and rats) were minced and incubated with a solution containing DNase (0.15 mg/mL), hyaluronidase type I-S (0.15 mg/mL), and collagenase type IA (1 mg/mL) (Sigma) in DMEM (Invitrogen, Carlsbad, CA) for 1 h at 37°C. Cells were washed and resuspended in complete media containing DMEM supplemented with 10% FBS (Invitrogen), glutamine (300 ng/mL), amphotericin B (250 μg/mL) (Sigma), and gentamicin (20 μg/mL) (Invitrogen). After overnight culture, non-adherent cells were removed and adherent cells were cultured. All experiments were performed with FLS after passage 4 (>95% FLS purity). All RA FLS cell lines were generated from orthopedic surgical specimens or ultrasound-guided synovial biopsies from rheumatoid factor-positive patients.

### Cell lysis, protein immunoprecipitation, and Western blots

FLS grown to 80% confluence were starved for 24 h followed by stimulation with PDGFβ 100 ng/mL (R&D Systems, Minneapolis, MN) for 30 min. The cells were washed with cold PBS, and resuspended in water containing protease and phosphatase inhibitors and subjected to three cycles of freezing (−20°C) and thawing. Total cell lysates were collected and immunoprecipitation was performed using Dynabeads™ M-270 Epoxy (Thermo Fisher Scientific) following the manufacturer’s instructions. Rabbit anti-rat-HIP1 antibody (20 μg) was used per sample (Millipore Sigma). Western blots with RA FLS cell extracts were done as previously described (20) and blotted with the following antibodies: anti-HIP1 (Abcam), anti-KIF1c (Abcam), anti-TRIOBP, anti-COPE, and anti-YWHAH (Abcam). Secondary fluorescent-labeled antibodies IRdye 800CW and IRDye 700 were used to detect the proteins (Licor, Lincoln, NE).

### Mass spectrometry

Dried frozen samples of total cell lysate with beads was sent to the Rockefeller University Proteomic Resource Center for processing and analyses. Proteins were eluted from beads using 8 M urea in 50 mM ammonium bicarbonate. Eluted proteins were reduced (DTT) and alkylated (iodoacetamide) followed by digestion (Endoproteinase LysC, Wako Chemicals and trypsin, Promega). Resulting peptides were desalted and analyzed by reversed-phase nano-LC-MS/MS (EasyLC, 1200 coupled to a Fusion Lumos, Thermo Scientific) (27). All data were quantified and searched against a UniProt rat database (July, 2014) using MaxQuant (v.1.5.3.28) (28) as well as Proteome Discoverer/Mascot. Oxidation of methionine and protein N-terminal acetylation were allowed as variable modifications, cysteine carbamidomethyl was set as a fixed modification, and two missed cleavages were allowed. “Match between runs” was enabled, and false discovery rates for proteins and peptides were set to 1%. Protein abundances were expressed as LFQ (label-free quantitation) values (29). Data were analyzed using Perseus (29). For the analyses, we excluded reverse hits and potential contaminants, and considered only proteins that were present in at least two of the three samples per strain. Missing values were inputted. Data were generated by the Proteomics Resource Center at the Rockefeller University.

### siRNA knockdown

Dharmacon SMARTpool siRNA targeting each gene, ubiquitin (housekeeping gene), or a non-coding control was purchased from Horizon (Lafayette, CO, USA) and transfected into DA or RA FLS according to the manufacturer’s instructions. Cells were then incubated at 37°C for 24–48 h prior to initiating any assay. Knockdown was confirmed with qPCR as previously described (**Supplementary Table 1**) (20, 30).

### Invasion assay

The *in vitro* invasiveness of FLS was assayed in a transwell system using Matrigel-coated inserts (BD Biosciences, Franklin Lakes, NJ), as previously described (12, 26). Briefly, 70%–80% confluent cells were harvested by trypsin-EDTA digestion, and resuspended in 500 µL of serum-free DMEM. A total of 2 × 10^4^ cells were placed in the upper compartment of each Matrigel-coated insert. The lower compartment was filled with media containing 10% FBS or RTK ligands and the plates were incubated at 37°C for 24 h. After 24 h, the upper surface of the insert was wiped with cotton swabs to remove non-invading cells and the Matrigel layer. The opposite side of the insert was stained with Crystal Violet (Sigma) and the total number of cells that invaded through Matrigel was counted at 25× magnification. Experiments were done in duplicate.

### Adhesion to Matrigel

Transfected cells were quickly trypsinized and counted. A total of 6,000 cells per well were plated in triplicate in a 96-well plate previously coated with 5 μg/mL of Matrigel (BD) in complete media. After 2 h, non-adherent cells were washed out with PBS 1× and adherent cells were stained with Crystal Violet. Cells were manually counted and read with a spectrophotometer at 590 nm.

### Wound healing (migration)

Transfected FLS were trypsinized and counted. A total of 6,000 cells per well were plated in triplicates in a 96-well plate. The cells were grown to confluence (usually 24 h), after which a wound was created by using a 10-µL tip. Pictures were taken at this time (time 0) and 24 h later. Cell migration was measured using ImageJ software by subtracting the density (pixels of the area covered by cells into wound area) after 24 h from the density (area covered by cells) of time point 0 (reference point).

### Proliferation

Transfected FLS were trypsinized and counted. A total of 3,000 cells per well were plated in triplicates in a 96-well plate in complete media. After the indicated times, cells were stained with Promega™ CellTiter 96™ AQueous One Solution Cell Proliferation Assay (MTS) (Madison, WI) following the manufacturer’s instructions. Proliferation was read at 490 nm.

### Rac1 activity

Rac1 activity was measured in cell lysates using the RAC1 G-ELISA Activation kit (Cytoskeleton, Denver, CO), according to the manufacturer’s instructions (20).

### Immunofluorescence microscopy

Immunofluorescence was performed as previously reported (26). Cells were treated with PDGF 100 ng/mL for 24 h. When indicated, cells were silenced for each target gene 24 h before PDGF treatment, fixed with 4% formaldehyde, permeabilized with PBS–Triton 0.01%, and stained with phalloidin (Thermo Scientific), pFAK (Thermo Scientific), anti-Kif1C (NovusBio Littleton, CO), anti-HIP (Millipore-Sigma), or anti-YWHAH (Abcam, Cambridge, MA). Cells were visualized and scored using a LEICA DMi8 microscope (Leica, Buffalo Grove, IL) with LASX software (Leica). The scoring of actin filaments, cell morphology, and lamellipodia was done as previously described (20). Briefly, four different RA FLS cell lines were used for the immunofluorescence analysis. Ten to fifty cells were analyzed per treatment for each RA FLS by taking random cell pictures. Cells were then scored and classified based on the following parameters: (a) actin filaments: 1 = no filaments visible in the central area of the cell, 2 = no thick filaments, but some fine filaments present in the central area of the cell, and 3 = >90% of the cell area filled with thick filaments; (b) cell morphology: 1 = round or stellar shape, 2 = elongated shape; (c) lamellipodia: considered positive if there was p-FAK staining on the cell peripheral structure, and scored as 1 = lamellipodia not present, 2 = distributed all around the cells, and 3 = lamellipodia polarized in one side of the cell (top or bottom or both).

### Statistics

We used the *t*-test to compare normally distributed data. One-way ANOVA was used in experiments where the same siRNA controls were used to compare against different gene-specific siRNA. The Fisher’s exact test was used to compare frequencies in contingency tables. All analyses (except for mass spectrometry) were done using the GraphPad Prism 10 software (Boston, MA). Volcano plot was constructed with VolcaNoseR (31).

## Results

### Mass spectrometry analyses identifies new HIP1-binding proteins

We used FLS obtained from arthritic DA and DA.ACI(R6) congenics rats (from here on referred to as “R6”). R6 is identical to DA except for the HIP1-contaning region where they differ in two SNPs in HIP1, including the predicted functional A749P. These two rat strains were used in the positional identification of HIP1 as a new arthritis gene (20). One FLS cell line per rat strain was analyzed in triplicate for mass spectrometry. The rat FLS-based strategy aimed at minimizing the potentially confounding effect of human HIP1 gene variants on its binding activity. There was an over-representation of GO biological processes, including those known to be regulated by HIP1, such as “Clathrin coat assembly”, “Regulation of clathrin-dependent endocytosis”, “Neurotransmitter receptor transport”, and “Regulation of platelet-derived growth factor receptor-beta (PDGFRb) signaling pathway” (**Table 1**, **Supplementary Table 2**). Additionally, others such as “Proteasome regulatory particle assembly”, “Isocitrate metabolic process”, “Glucocorticoid receptor signaling pathway”, “Dendritic transport”, and “Regulation of mRNA stability involved in response to stress” were new findings in association with HIP1-binding proteins (**Table 1**, **Supplementary Table 2**).

**Table 1:** Selected list of GO biological processes overrepresented among the HIP1-binding proteins.

GO term ID	GO term description	Observed protein count	Background protein count	Strength	FDR	Matching proteins in the network
GO:0006177	GMP biosynthetic process	4	4	1.28	0.0065	HPRT1, IMPDH2, IMPDH1, GMPS
GO:0030043	Actin filament fragmentation	4	4	1.28	0.0065	DSTN, CFL2, WDR1, CFL1
GO:0010610	Regulation of mRNA stability involved in response to stress	3	3	1.28	0.031	IGF2BP1, MYEF2, HNRNPM
GO:0070682	Proteasome regulatory particle assembly	3	3	1.28	0.031	tRIM21, UBXN1, PARK7
GO:0006102	Isocitrate metabolic process	5	6	1.2	0.0022	ACO2, IDH3A, IDH2, IDH3B, IDH1
GO:0071816	Tail-anchored membrane protein insertion into ER membrane	5	6	1.2	0.0022	SGTA, GET4, UBL4A, BAG6, ASNA1
GO:0042921	Glucocorticoid receptor signaling pathway	4	5	1.18	0.0104	NR3C1, YWHAH, CALR, NEDD4
GO:0090086	Negative regulation of protein deubiquitination	3	4	1.15	0.0479	TRIM21, UBXN1, PARK7
GO:0090168	Golgi reassembly	3	4	1.15	0.0479	VCPIP1, PDCD10, YWHAZ
GO:1902975	Mitotic DNA replication initiation	3	4	1.15	0.0479	MCM4, MCM2, MCM3
GO:0048268	**Clathrin coat assembly**	11	18	1.06	2.32E-06	CLTA, HIP1R, AP2S1, PIK3C2A, CLTB, HIP1, EPS15, PICALM, CLINT1, CLTC, AP2B1
GO:0045899	Positive regulation of RNA polymerase II transcription preinitiation complex assembly	6	10	1.05	0.0017	PSMC4, PSMC5, PSMC2, PSMC6, CAND1, PSMC3
GO:0043248	Proteasome assembly	8	14	1.03	0.00016	PSMD5, PSMD10, PSMD11, PSMD4, POMP, PSMD13, PSMD9, ADRM1
GO:0000727	Double-strand break repair via break-induced replication	6	11	1.01	0.0023	MCM5, MCM4, MCM6, MCM2, MCM7, MCM3
GO:0070142	Synaptic vesicle budding	5	10	0.98	0.0101	AP3B1, AP3D1, DNM1, DNM2, PICALM
GO:0098935	Dendritic transport	5	10	0.98	0.0101	HNRNPU, KIF5B, PURA, FLOT2, KIF5A
GO:0061684	Chaperone-mediated autophagy	4	8	0.98	0.0311	HSP90AA1, ATG7, BAG3, LAMP2
GO:0098884	Postsynaptic neurotransmitter receptor internalization	4	8	0.98	0.0311	EPS15, DNM1, DNM2, AP2B1
GO:2000586	**Regulation of platelet-derived growth factor receptor-beta signaling**	4	8	0.98	0.0311	LOX, LRP1, HIP1R, HIP1
GO:0022417	Protein maturation by protein folding	4	9	0.92	0.0411	AIP, CALR, PRDX4, FKBP1A
GO:0043653	Mitochondrial fragmentation involved in apoptotic process	4	9	0.92	0.0411	FIS1, VPS35, CCAR2, DNM1L
GO:0099641	Anterograde axonal protein transport	4	9	0.92	0.0411	HSPB1, MAP1A, KIF5B, KIF5A
GO:0038061	NIK/NF-kappaB signaling	35	81	0.91	2.44E-16	PSMA4, PSMC4, PSMD5, PSMD8, PSMA3, PSME2, PSMD10, PSMD7, PSMA2, NFKB1, SKP1, PSMC1, PSMA6, PSMD11, PSMB6, PSMA5, PPP4C, PSMD1, PSMD2, PSMC5, PSMF1, COPS8, PSMD4, PSMA7, PSME1, RELA, PSMD14, PSMC2, PSMD13, PSMC6, PSMA1, PSMD6, PSMD9, AKT1, PSMC3
GO:0032801	Receptor catabolic process	11	26	0.9	3.39E-05	CLTA, AP2S1, AP2M1, AP2A2, AP2A1, LGMN, NEDD4, CAPN1, CLTC, SH3GLB1, AP2B1
GO:2000369	**Regulation of clathrin-dependent endocytosis**	7	17	0.89	0.0025	HIP1R, DAB2, BMP2K, UBQLN2, SMAP1, DNM2, AAK1
GO:0034063	Stress granule assembly	8	20	0.88	0.001	EIF2S1, PUM2, DYNC1H1, G3BP2, G3BP1, UBAP2L, LSM14A, CSDE1
GO:0072583	**Clathrin-dependent endocytosis**	11	29	0.86	7.57E-05	CLTA, AP2S1, AP2M1, CLTB, GAK, AP2A2, HIP1, AP2A1, PICALM, CLTC, AP2B1

**Boldface** denotes pathways known to involve HIP1.

### HIP1 amino-acid differences interfere with its binding to other proteins

**Figure 1:**
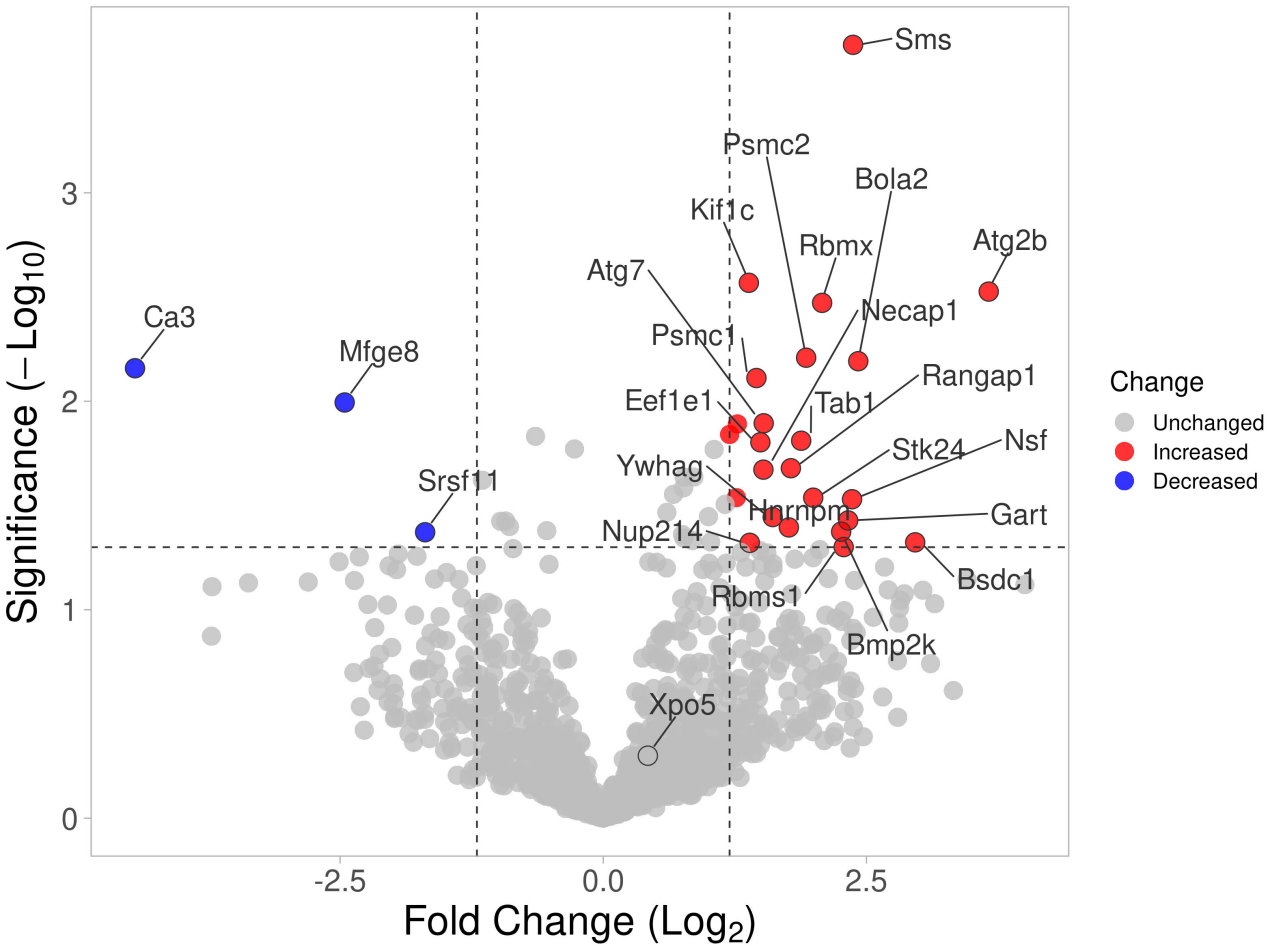
Volcano plot of all proteins identified by mass spectrometry. Proteins with significantly increased detection using cell extracts from arthritic DA FLS are shown in red, and those detected in reduced levels compared with protected R6 rats (arthritis-resistant ACI alleles at HIP1) are shown in blue.

A total of 45 HIP1-binding proteins had significantly different levels in DA and R6 (**Figure 1**). These proteins had functions that included endocytosis (KIF1C, NECAP1, and SLC9a3r1), cytoskeletal and cell movement (AAMP, CCT8, ENO1, KIF1C, KANK2, MFGE8, TRIOBP, and VCL), kinase and signal transduction (TAB1, and the 14-3-3 proteins YWHAG and YWHAH), autophagy (ATG2b), and others (**Table 2**).

**Table 2:** New HIP1-interacting proteins and their functional characteristics in rheumatoid arthritis fibroblast-like synoviocytes.

			Effect of the siRNA knockdown in RA FLS (*n* = 3-7 per experiment)*				
Symbol	Protein name	Human	Proliferation	Adhesion	Wound healing	Invasion	IP + WB**
AAMP	angio associated migratory cell protein	ENSG00000127837	N	N	N	N	
AP3BI	AP-3 complex subunit beta-1	ENSG00000132842	N	N	N	N	
ATG2B	Autophagy-Related Protein 2 Homolog B	ENSG00000066739	N	N	N	N	
BMP2K	BMP-2-Inducible Protein Kinase	ENSG00000138756	N	N	N	N	
BOLA2	bolA family member 2	ENSG00000183336	N	N	N	N	
BSDC1	BSD Domain Containing 1	ENSG00000160058	N	**H (*p* = 0.007)**	N	N	
CA3	Carbonic anhydrase 3	ENSG00000164879	N	N		**L (*p* = 0.0165)**	
CCT2	T-complex protein 1 subunit beta	ENSG00000166226	N	N	N	N	
CCT8	chaperonin containing TCP1 subunit 8	ENSG00000156261	N	N	N	N	
CHMP4BL1/CHAMP	chromatin modifying protein 4B-like 1	ENSG00000101421	N	N	N	**L (*p* = 0.0246)**	
COMMD9	COMM domain containing 9	ENSG00000110442	N	N	N	N	
COPE	coatomer protein complex subunit epsilon	ENSG00000105669	N	N	N	**L (*p* = 0.0441)**	+
EEFIEI	Eukaryotic translation elongation factor 1 epsilon-1	ENSG00000124802	N	N	N	N	
ENO1	Alpha-enolase	ENSG00000074800	N	**H (*p* = 0.01)**	N	N	
GART	phosphoribosylglycinamide formyltransferase, phosphoribosylglycinamide synthetase, phosphoribosylaminoimidazole synthetase	ENSG00000159131	N	N	N	N	
KANK2	KN motif and ankyrin repeat domains 2	ENSG00000197256	N	N	N	N	
KIF1C	Kinesin-like protein;Kinesin-like protein KIF1C	ENSG00000129250	N	N	**L (*p* = 0.04)**	**L (*p* = 0.02)**	+
MFGE8	Lactadherin	ENSG00000140545	N	N	N	N	
NECAP1	Adaptin ear-binding coat-associated protein 1	ENSG00000089818	N	N	N	N	
NUP214	nucleoporin 214	ENSG00000126883	N	N	N	N	
PRRC1	Protein PRRC1	ENSG00000164244	N	N	N	N	
PSMC1	26S protease regulatory subunit 4	ENSG00000100764	N	N	N	N	
RANGAP1	Ran GTPase activating protein 1	ENSG00000100401	**L (*p* = 0.05)**	N	N	N	
RBMX	RNA-binding motif protein, X chromosome retrogene-like;	ENSG00000213516	N	N	N	N	
Slc9a3r1	Na(+)/H(+) exchange regulatory cofactor NHE-RF1	ENSG00000109062	N	N	N	N	
SMS	Spermine synthase	ENSG00000102172	N	N	N	N	
STK24	Serine/threonine-protein kinase 24	ENSG00000102572	N	N	N	N	
TAB1	TGF-Beta Activated Kinase 1 (MAP3K7) Binding Protein 1	ENSG00000100324	N	**H (*p* = 0.02)**	N	N	
TRIOBP	TRIO and F-actin binding protein	ENSG00000100106	N	N	N	N	+
VCL	Vinculin	ENSG00000035403	N	N	N	N	
YWHAG	14-3-3 protein gamma; tyrosine 3-monooxygenase/tryptophan 5-monooxygenase activation protein, gamma	ENSG00000170027	N	N	**L (*p* = 0.02)**	**L (*p* = 0.0196)**	
YWHAH	14-3-3 protein; tyrosine 3-monooxygenase/tryptophan 5-monooxygenase activation protein, eta eta	ENSG00000128245	N	N	**L (*p* = 0.05)**	**L (*p* = 0.04)**	+

**H, higher levels; L, lower levels; *four of the proteins were examined.

Green, H (higher); Red, L (lower).

Eleven proteins were detected only in the anti-HIP1 immunoprecipitates from DA, and two only in the R6 arthritis-resistant anti-HIP1 immunoprecipitates. Thirteen had increased bound protein levels to DA, while five had increased binding to R6 (**Supplementary Table 3**). Levels of most of the HIP1-binding proteins identified by mass spectrometry were not significantly different between DA and R6, suggesting that their binding was not affected by the presence of the HIP1 amino-acid changing SNP (20).

### Confirmation of HIP1 binding in human RA FLS

**Figure 2:**
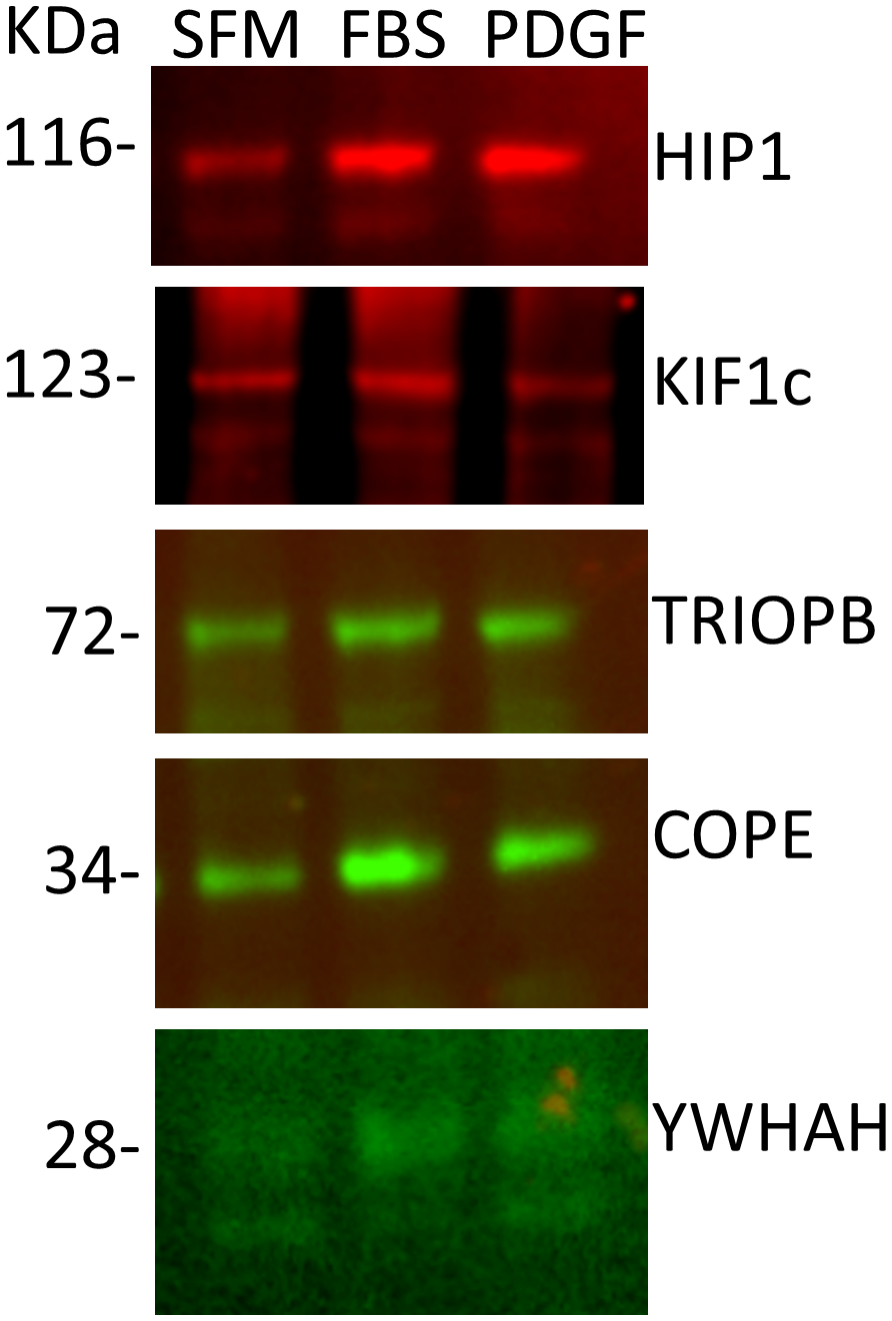
Western blots of five selected proteins confirm their binding to HIP1. RA FLS cell lines were cultured in serum-free media (SFM), media with FBS, or media with PDGF 100 ng/mL for 24 h, followed by cell lysis, immunoprecipitation with anti-HIP1 antibodies, and Western blot with the protein-specific antibodies confirming that KIF1C, TRIOBP, COPE, and YWHAH bind to HIP1 (10 μL of 1 μg/μL of protein lysate per lane).

In order to validate the mass spectrometry discoveries, we cultured human FLS cell lines generated from patients with RA (20, 32) and used their cell lysates for immunoprecipitation with anti-HIP1 antibody, followed by Western blot with anti-COPE, anti-KIF1C, anti-TRIOBP, and anti-YWHAH. All four proteins were identified in the anti-HIP1 immunoprecipitates, confirming their binding to human HIP1 (**Figure 2**).

### Knockdown of selected new HIP1-binding proteins interferes with RA FLS cell phenotypes

Of the 45 significantly different genes in the mass spectrometry analyses in rat FLS, 32 were selected for studies in RA FLS (**Table 2**, **Supplementary Table 3**) based on their known function, potential relevance to FLS, and the availability of reagents for protein analyses. RA FLS were transfected with siRNA to knock down each one of these 32 genes, followed by qPCR confirmation of the knockdown (**Supplementary Figure 1**).

**Figure 3:**
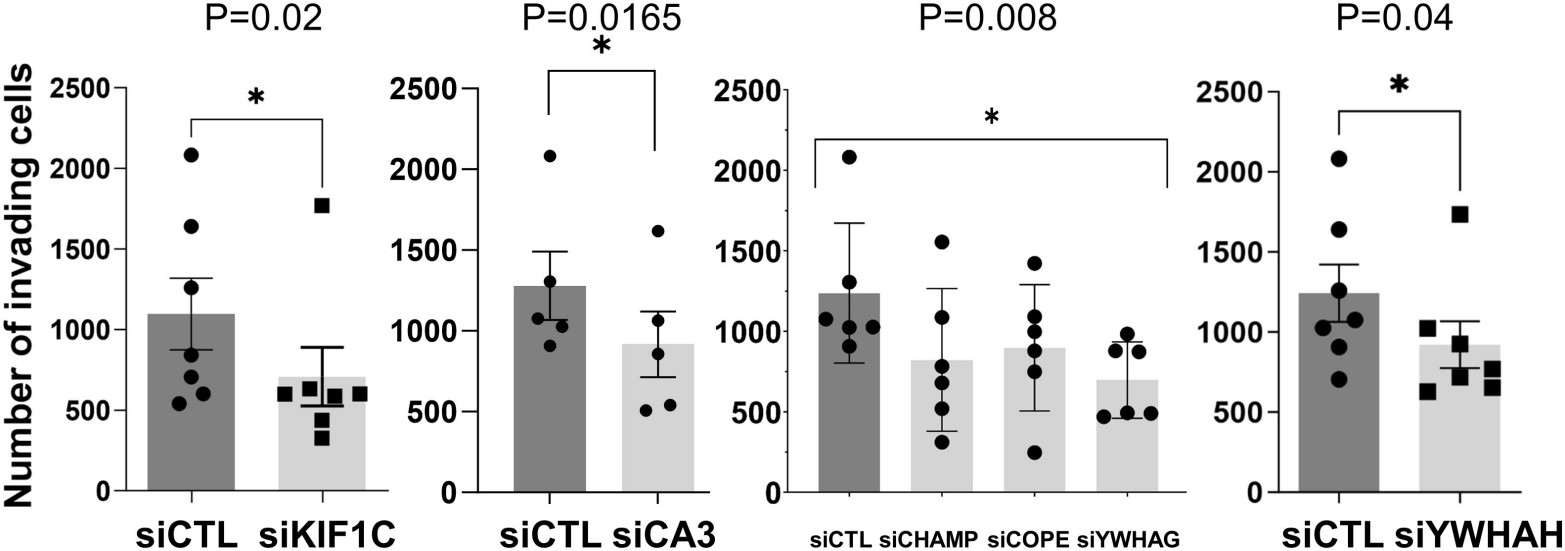
siRNA knockdown of six genes decreased RA FLS invasiveness. FLS from six to eight different patients with RA were transfected with siRNA control or siRNA specific for each gene (CA3, CHAMP3, COPE, KIF1C, YHHAG, and YWHAH) and tested for invasiveness through Matrigel-covered inserts over a 24-h period (mean ± SEM, *p-value < 0.05 as shown in the figure; KIF1C, CA3, and YWHAH were analyzed with a paired *t*-test, and CHAMP, COPE, and YWHAG were analyzed with one-way ANOVA).

Knockdown of 10 out of the 32 genes induced significant changes in the RA FLS phenotypes (**Table 2** and **Supplementary Table 3**). Specifically, knockdown of five different HIP1-binding protein genes (CHMP4BL1, COPE, KIF1C, YWHAG, and YWHAH) significantly decreased FLS invasiveness (**Figure 3**). None of the other genes affected RA FLS invasion (**Supplementary Figure 2**). CA3 induced cell death and therefore was not considered to reduce invasiveness *per se*.

**Figure 4:**
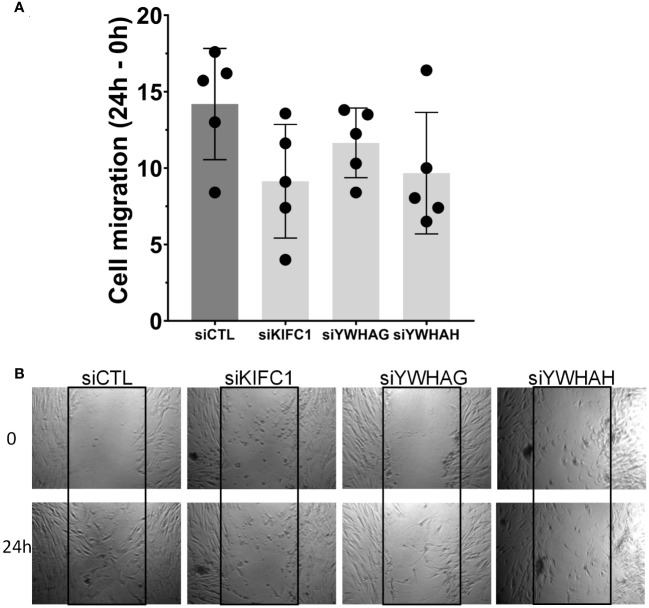
Knockdown of three genes (KIF1C, YWHAG, and YWHAH) decreased RA FLS cell migration in the wound healing (scratch assay). **(A)** FLS transfected siRNA specific for each gene had their migration reduced over a 24-h period, compared with siRNA control (five different RA FLS cell lines per treatment condition, run in triplicate; mean ± SEM; one-way ANOVA, *p*-value = 0.0937, not significant). **(B)** Representative images at 0 h and 24 h; boxes identify the space quantified (scale bar = 200 μm).

Knockdown of KIF1C, YWHAG, and YWHAH also reduced RA FLS migration in the wound healing assay (**Figures 4A, B**), consistent with the observed reduction in invasiveness. None of the other genes affected RA FLS migration in the wound healing assay (**Supplementary Figure 3**).

**Figure 5:**
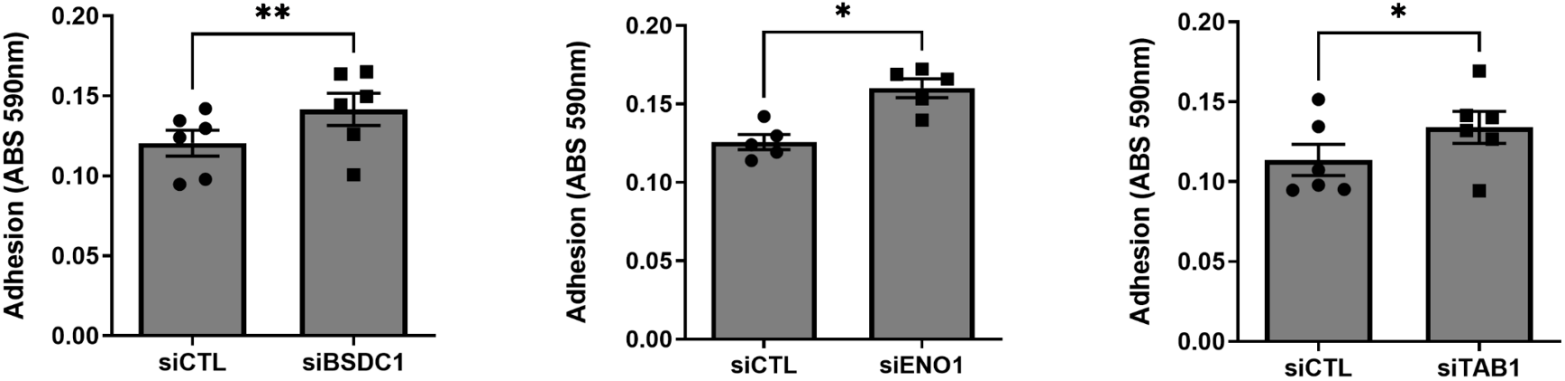
Knockdown of three genes increased RA FLS cell adhesion. FLS transfected siRNA specific for BSDC1, ENO1, and TAB1 genes had their adhesion significantly increased after a 2-h period, compared with siRNA control (five to six different RA FLS cell lines per treatment condition; mean ± SEM, *p-value < 0.05, **p-value < 0.01, paired *t*-test).

Knockdown of BSDC1, ENO1, and TAB1 increased RA FLS adhesion (**Figure 5**) but had no detectable effect on the other phenotypes. RANGAP1 knockdown reduced FLS proliferation (**Supplementary Figure 4**) without affecting any other phenotype. None of the other genes affected RA FLS proliferation or adhesion (**Supplementary Figures 4**, **5**).

### RA FLS stimulation with PDGF increased KIF1C localization to the lamellipodia

**Figure 6:**
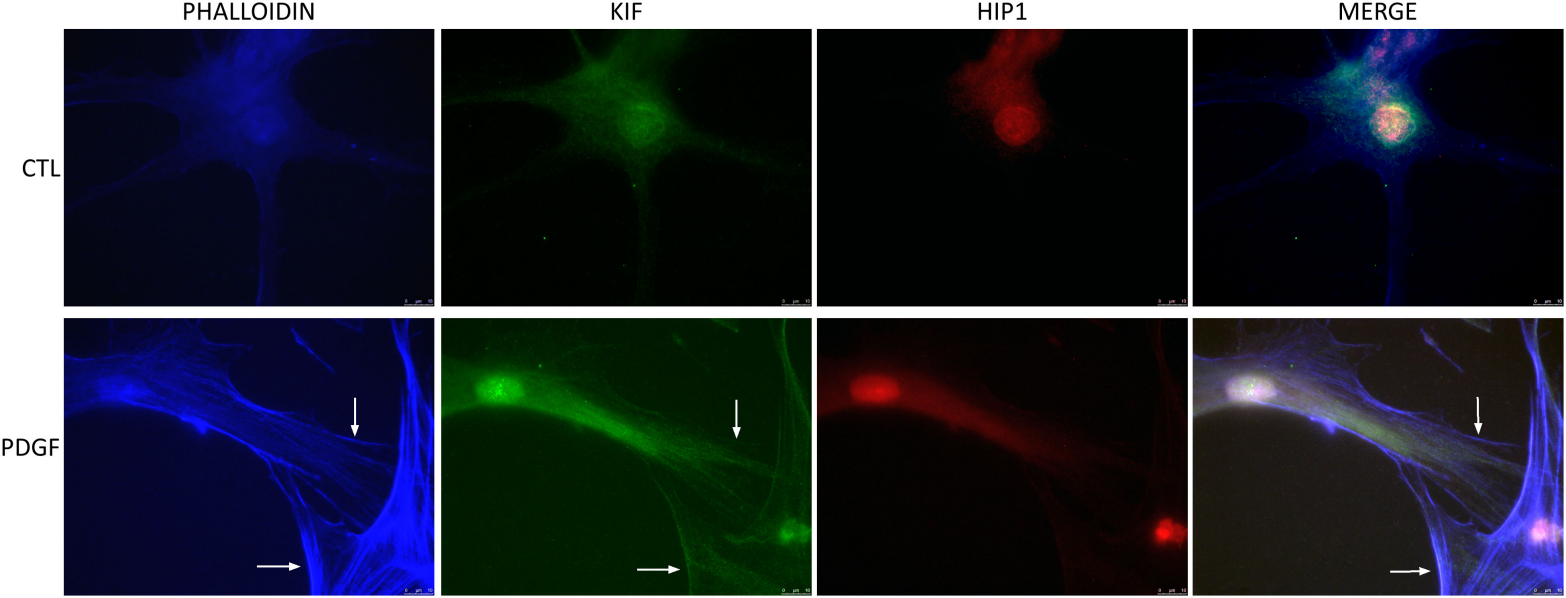
KIF1C localization in resting and activated RA FLS. FLS were cultured for 24 h in serum-free media (controls) or PDGFβ 100 ng/mL and stained for actin (phalloidin blue), KIF1C (green), or HIP1 (red). There was increased presence of KIF1C in lamellipodia of activated cells (arrows identify the lamellipodia; representative images of immunofluorescence analyses; scale bars = 10 μm).

Given the effect of KIF1C in RA FLS invasion and migration, we next examined its cell localization. KIF1C was predominantly present in the cytosol. Following PDGF stimulation, there was increased KIF1C localization in the lamellipodia (**Figure 6**). HIP1 colocalized with KIF1C in the cytosol, but not in lamellipodia, suggesting different cellular roles in the regulation of FLS functions (**Figure 6**).

### KIF1C knockdown interferes with FLS morphology and actin cytoskeleton

**Figure 7:**
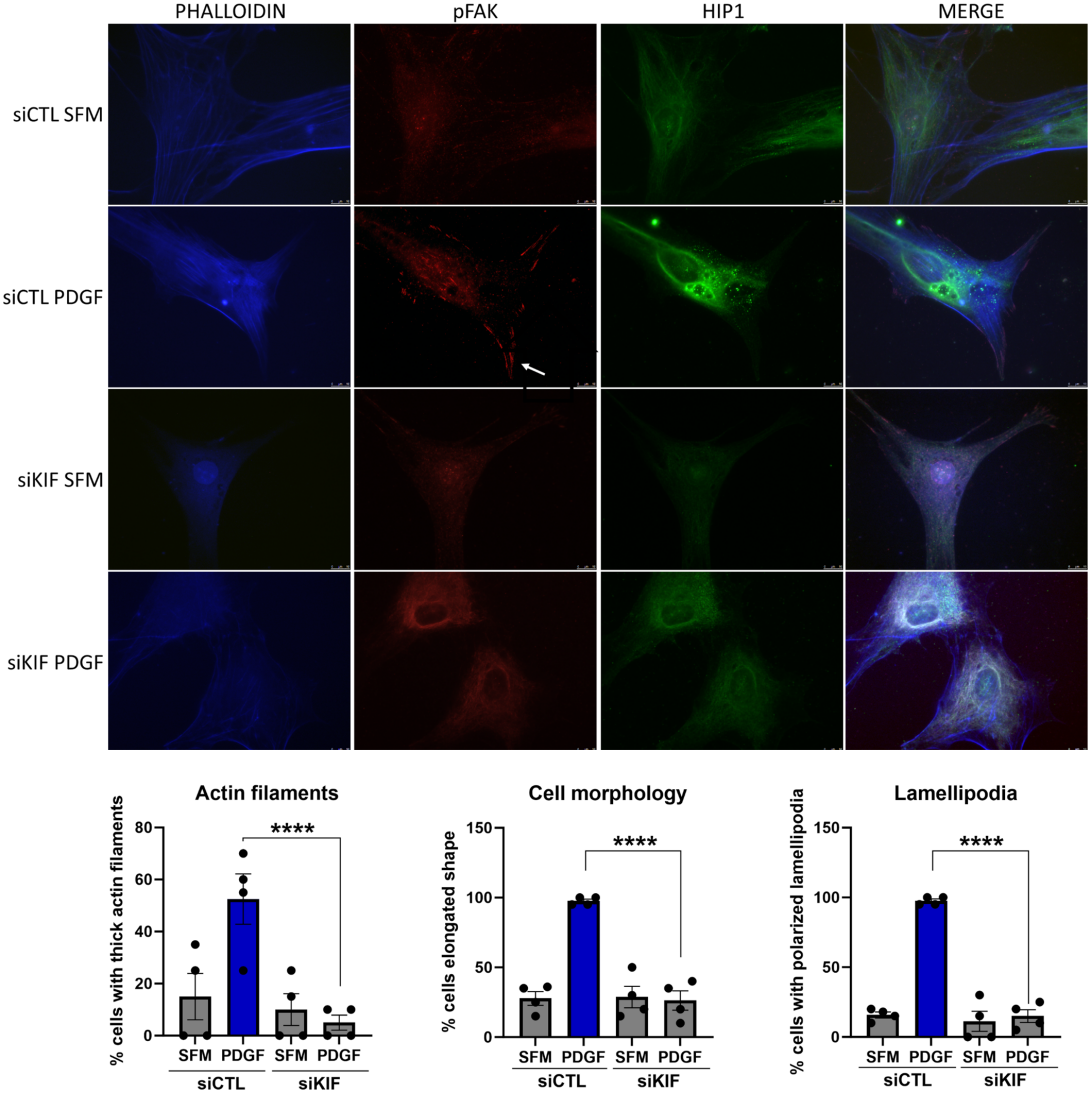
Immunofluorescence analyses of the effect of KIF1C knockdown on RA FLS morphology. PDGFβ 100 ng/mL increased the numbers of thick actin filaments, the number of elongated cells, and the number of pFAK staining lamellipodia (arrow). siRNA knockdown of KIF1C prevented the above changes (RA FLS cell lines from four different patients were analyses; 15–20 cells per RA FLS cell lines were scored; ****P < 0.0001, Fisher’s exact test; scale bars = 10 μm).

Knockdown of KIF1C not only induced the most significant reduction in FLS invasiveness but also induced changes in FLS morphology, with cells developing a more round or stellate shape with disorganized and thin actin fibers, significantly different from siRNA controls (**Figure 7**). KIF1C knockdown also reduced the number of lamellipodia and their colocalization with pFAK, but did not affect the localization of HIP1 in the cell (**Figure 7**).

**Figure 8:**
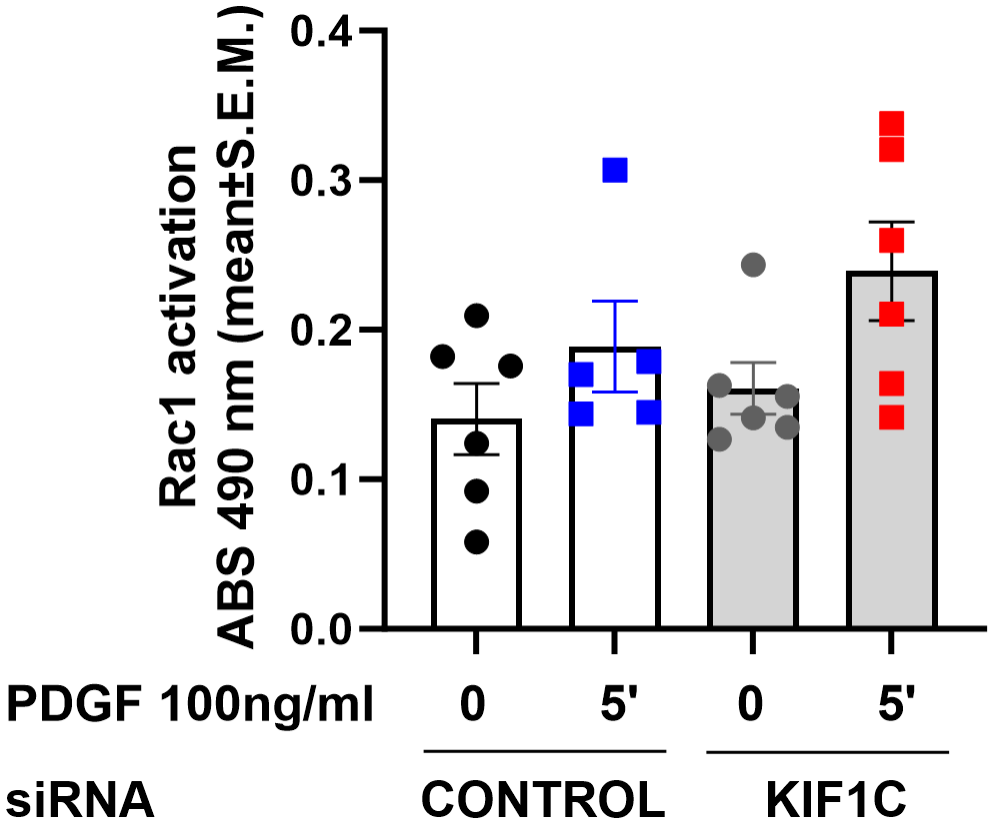
Effect of KIF1C knockdown on Rac1 activation. siRNA knockdown of KIF1C did not interfere with PDGFβ-induced (5 min) RAC1 activation. There was no statistically significant difference between siRNA KIF1C and siRNA control groups.

Given that HIP1 binds to KIF1C, and the FLS phenotypic similarities between knockdown of both genes, we next examined whether KIF1C was also involved in the regulation of Rac1 activity. However, KIF1C knockdown had no significant suppressive effect on PDGF-induced Rac1 activation (**Figure 8**), suggesting different roles or functions in the regulation of FLS invasiveness.

## Discussion

We have previously reported the discovery of a new arthritis severity gene, HIP1, in studies in mice and rats (20, 30). A HIP1 SNP regulates arthritis severity in pristane-induced arthritis in rats, and knockdown of HIP1 reduced the invasive properties of FLS from arthritic rats and patients with RA, which is a clinically relevant *in vitro* phenotype that strongly correlates with histological and radiographic damage in rodent models of arthritis (12) and in RA (19). These observations suggested that variants within the HIP1 gene affected its function and perhaps its ability to bind to other proteins to activate FLS invasion processes. However, little was known about possible HIP1-binding proteins.

In the present study, we used the functional differences associated with the A749P HIP1 SNP in DA and R6 rats, and used an anti-HIP1 to immunoprecipitate HIP1-binding proteins. A number of proteins were identified in the immunoprecipitate, including some that had been previously reported to bind HIP1 such as HIP1R, clathrin, and actin (33, 34). We focused our analyses on the proteins differentially detected in DA and R6 as we considered that those were more likely to affect HIP1-regulated FLS phenotypes. Based on the protein cell localization or existing functional information, 31 of the 45 differentially detected proteins were selected for functional studies and siRNA knockdown. Knockdown of 10 of these genes (nearly 30%) induced significant changes in the RA FLS phenotypes, including invasiveness, migration, adhesion, and cell proliferation, underscoring the role of HIP1 and its binding protein in the regulation of these cellular phenotypes.

KIF1C was of particular interest as knockdown of this gene induced the most significant reduction in RA FLS invasion. We confirmed KIF1C binding to human HIP1, and knockdown of KIF1C changed cell morphology from a typically fusiform to a round or stellate shape, with reduced formation of lamellipodia, which are structures required for cell motility and invasion. However, while KIF1C was present in increased levels in lamellipodia of activated FLS, HIP1 was not. Furthermore, unlike HIP1, KIF1C knockdown did not affect PDGF-induced Rac1 activation after PDGF stimulation for 5 min (the time point that was previously shown to detect the most significant HIP1-mediated Rac1 activation in RA FLS).

These observations suggest that while KIF1C and HIP1 bind to each other and are required for FLS invasiveness and mobility, they have different roles in the invasive processes where HIP1 regulates PDGFR signaling, while KIF1C may be more directly involved in the processes taking place in the lamellipodia itself (20). To our knowledge, this is the first time that KIF1C is detected in FLS, and the first time that it is studied in the context of arthritis and autoimmune rheumatic diseases. We did find one previous report implicating KIF1C mutations in an experimental model of autoimmune oophoritis, though the precise mechanism of action was not completely clear (35).

KIF1c is part of the kinesin family of protein transporters (36). Kinesins have a central role in cellular membrane trafficking and are essential for the function of many polar cells, such as neurons, epithelial cells, or stem cells during organogenesis (36, 37). Kinesins also play a key role in cell-cycle dynamics (36, 38) and in cancer cells, where they have been associated with tumor growth and metastasis (39, 40). KIF1c enables protein binding to microtubules, mRNA binding, and cytoskeleton changes (41, 42). In cancer cells, KIF1c can be activated by c-Src to mediate the formation of invadopodia, required for cell invasion (43). KIF1c mRNA is also present in increased levels in invadopodia where it guides partnering proteins to interact (42). Therefore, it is conceivable that the PDGFR activation and binding to HIP1 increases HIP1 binding to and expression of KIF1c, and together contribute to actin cytoskeleton reorganization, transporting KIF1c and other proteins to the invadopodia and lamellipodia as we show in the present study, to enhance FLS mobility, invasion, and, ultimately, joint damage.

Interestingly, KIF1c is also known to bind to other proteins such as 14-3-3 proteins (44). YWHAG, YWHAH, and others are part of the 14-3-3 proteins and were immunoprecipitated with anti-HIP1. While 14-3-3 proteins binding to HIP1 was confirmed by Western blot, it is conceivable that the immunoprecipitation of 14-3-3 proteins with anti-HIP1 may have been secondary to their binding to KIF1c. Nearly 200 14-3-3 proteins have been reported (45). These proteins can bind to and interfere with the activity of transcription factors such as NFκB, STATs, and PPAR, and interfere with TLR receptor-activated immune responses (46). Some 14-3-3 proteins may have anti-inflammatory activity while others have pro-inflammatory activity (46). 14-3-3eta (YWHAH) has been identified in the serum and synovial fluid of patients with RA, and levels correlate with disease severity and activity (47–49). 14-3-3zeta (YWHAZ) protein was shown to have a suppressive activity in arthritis in rats, and its deletion increased disease severity (50). 14-3-3gamma (YWHAG) and 14-3-3eta (YWHAH) were among the proteins co-immunoprecipitated with anti-HIP1, and their knockdown significantly reduced FLS adhesion and invasion. Taken together, it is conceivable that the multiple 14-3-3 proteins co-immunoprecipitated with HIP1, perhaps indirectly via KIF1c binding, together have a pro-invasion and, therefore, pro-joint damaging properties.

In conclusion, we describe the identification of several new HIP1-binding proteins and a comprehensive characterization of the function of 31 of those in RA FLS. These studies led to the identification of 10 new proteins involved in the regulation of relevant FLS phenotypes including the highly relevant invasiveness, including KIF1C.

## Data availability statement

The original contributions presented in the study are publicly available. This data can be found here: Proteome Xchange, PXD051384 (https://www.ebi.ac.uk/pride/archive/projects/PXD051384).

## Ethics statement

The studies were conducted in accordance with the local legislation and institutional requirements. The patients/participants provided written informed consent to participate in studies using their tissues and cells. The samples used in this study were approved from the following: Cell lines generated from tissues obtained by the FITDP had Feinstein Institute IRB approval. Cell lines generated from Synovial biopsies at Mount Sinai School of Medicine under an IRB-approved protocol. Synovial cell lines from the HSS and UCSD were de-identified and had approval from the respective institution’s IRB.

## Author contributions

TL: Conceptualization, Data curation, Formal Analysis, Investigation, Methodology, Project administration, Supervision, Validation, Writing – original draft, Writing – review & editing. CH: Data curation, Formal Analysis, Investigation, Methodology, Writing – review & editing. PG: Conceptualization, Data curation, Formal Analysis, Funding acquisition, Investigation, Methodology, Project administration, Resources, Supervision, Visualization, Writing – original draft, Writing – review & editing.

## Supplementary material

Supplementary Figure 1 qPCR confirmation of gene knockdown. The knockdown of all genes selected and studied in functional assays was confirmed by qPCR and shown as fold difference. Blue box highlights that six genes that were also associated with reduced RA FLS invasiveness.

Supplementary Figure 2 Knockdown of twenty-three genes did not significantly affect RA FLS invasiveness (1-6 different cell lines per treatment condition; shown as mean ± SEM).

Supplementary Figure 3 Knockdown of twenty-eight genes did not significantly affect RA FLS mobility in the wound healing (scratch) assay (2-6 different cell lines per treatment condition; shown as mean ± SEM).

Supplementary Figure 4 Only one (RANGAP1) of the thirty-one genes tested significantly affected RA FLS proliferation. Knockdown of only one gene (marked with a square) significantly affected (reduced) RA FLS proliferation (*P ≤ 0.05), (2-7 different cell lines per treatment condition; shown as mean ± SEM).

Supplementary Figure 5 Adhesion assay of twenty-seven genes. None of these genes significantly affected RA FLS adhesion (2-6 different cell lines per treatment condition; shown as mean ± SEM).

Supplementary Figure 6 Protein from cell extracts from DA and R6 were quantified and identical amounts loaded into a protein gel for electrophoresis (10μl of a 1μg/μl solution) followed by staining with Commassie blue. HIP1 is predicted to migrate into the position 116.
