# Supplementary figures and images for: KIF1C and new Huntingtin-interacting protein 1 binding proteins regulate rheumatoid arthritis fibroblast-like synoviocytes’ phenotypes
Source: Front Immunol. 2024 Apr 25;15:1323410. doi: 10.3389/fimmu.2024.1323410 (PMC11079228; doi:10.3389/fimmu.2024.1323410)

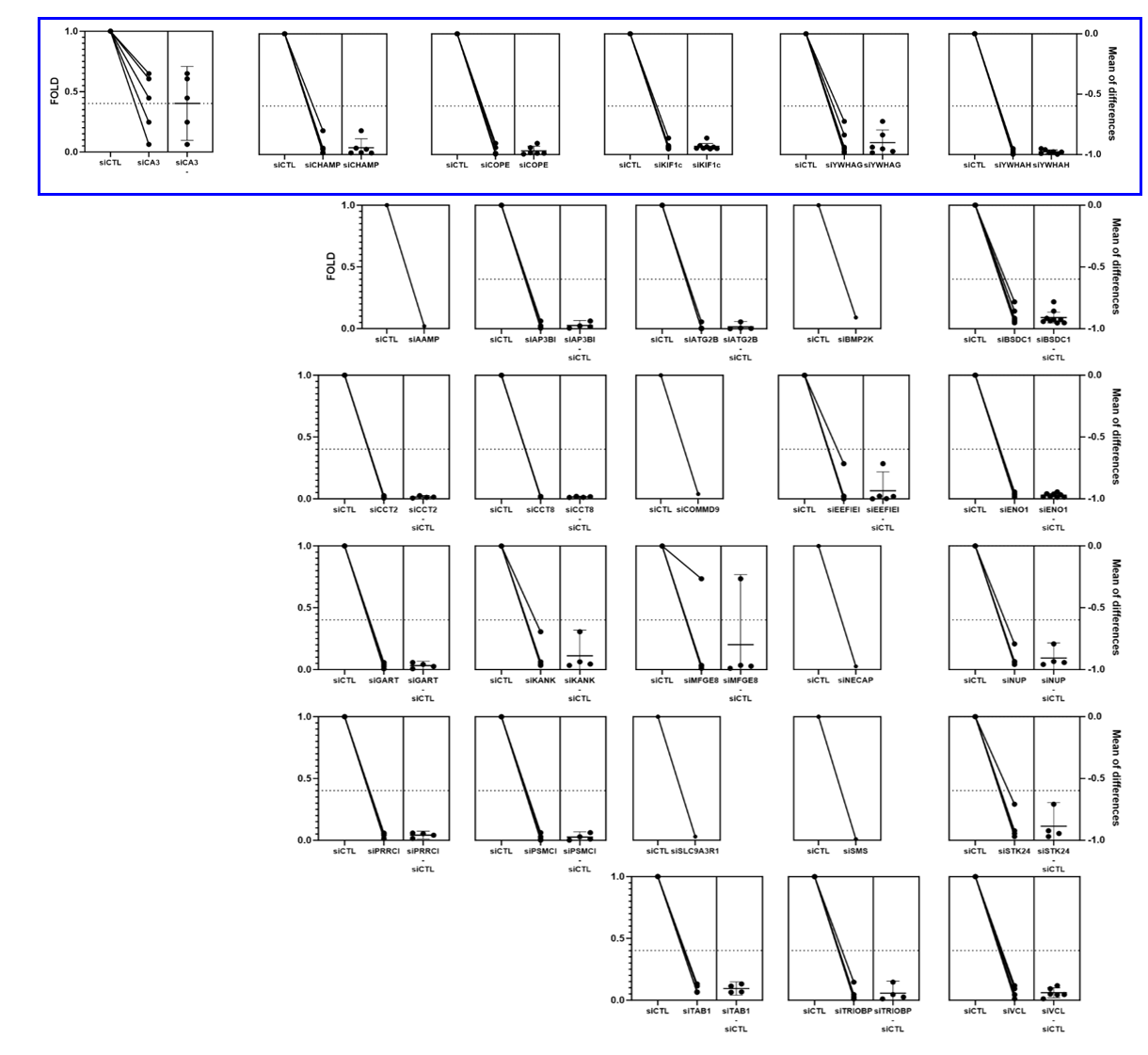

Supplement: Supplementary Figure 1 — qPCR confirmation of gene knockdown. The knockdown of all genes selected and studied in functional assays was confirmed by qPCR and shown as fold difference. Blue box highlights that six genes that were also associated with reduced RA FLS invasiveness. [file Image_1.tif]

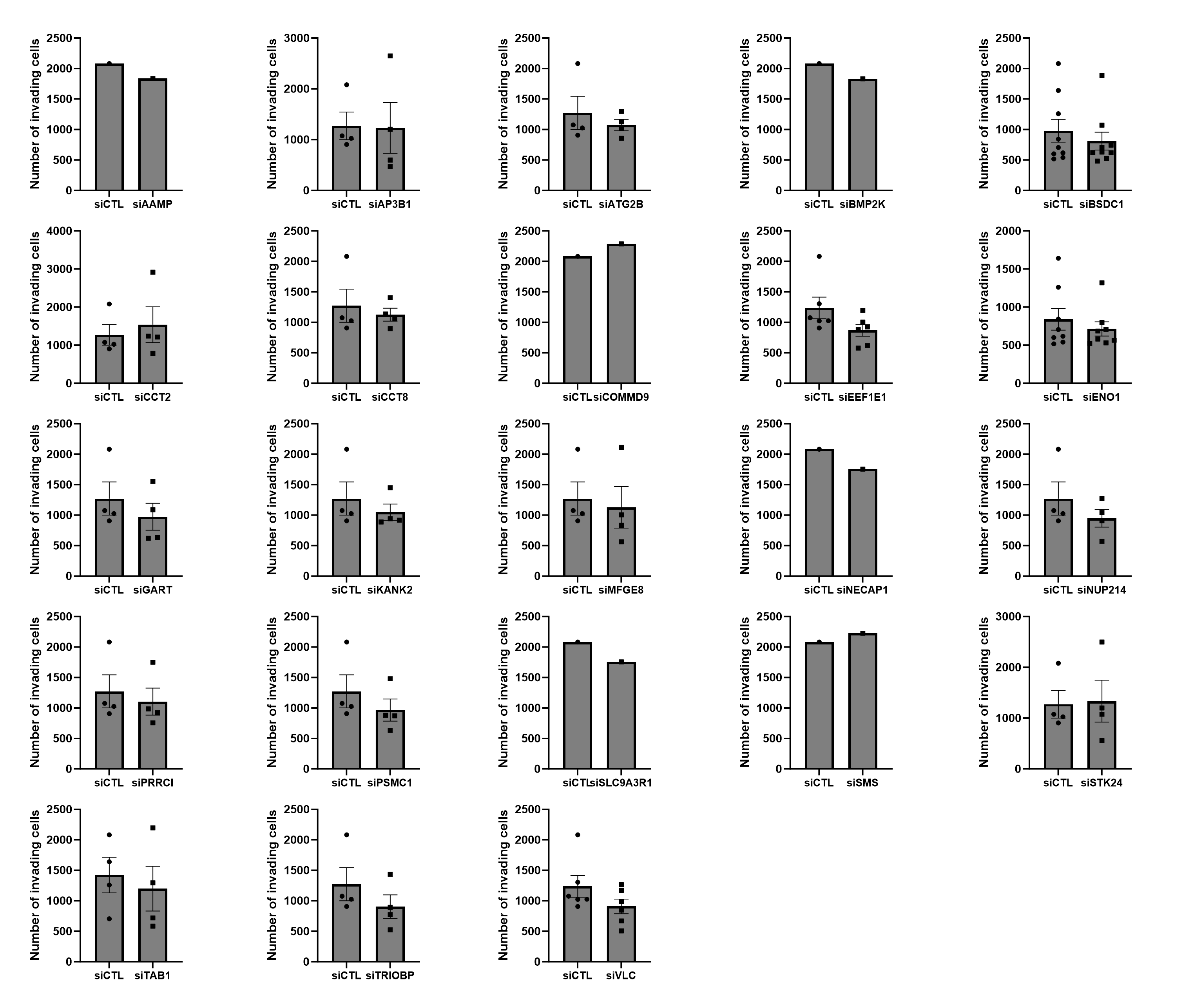

Supplement: Supplementary Figure 2 — Knockdown of twenty-three genes did not significantly affect RA FLS invasiveness (1-6 different cell lines per treatment condition; shown as mean ± SEM). [file Image_2.tif]

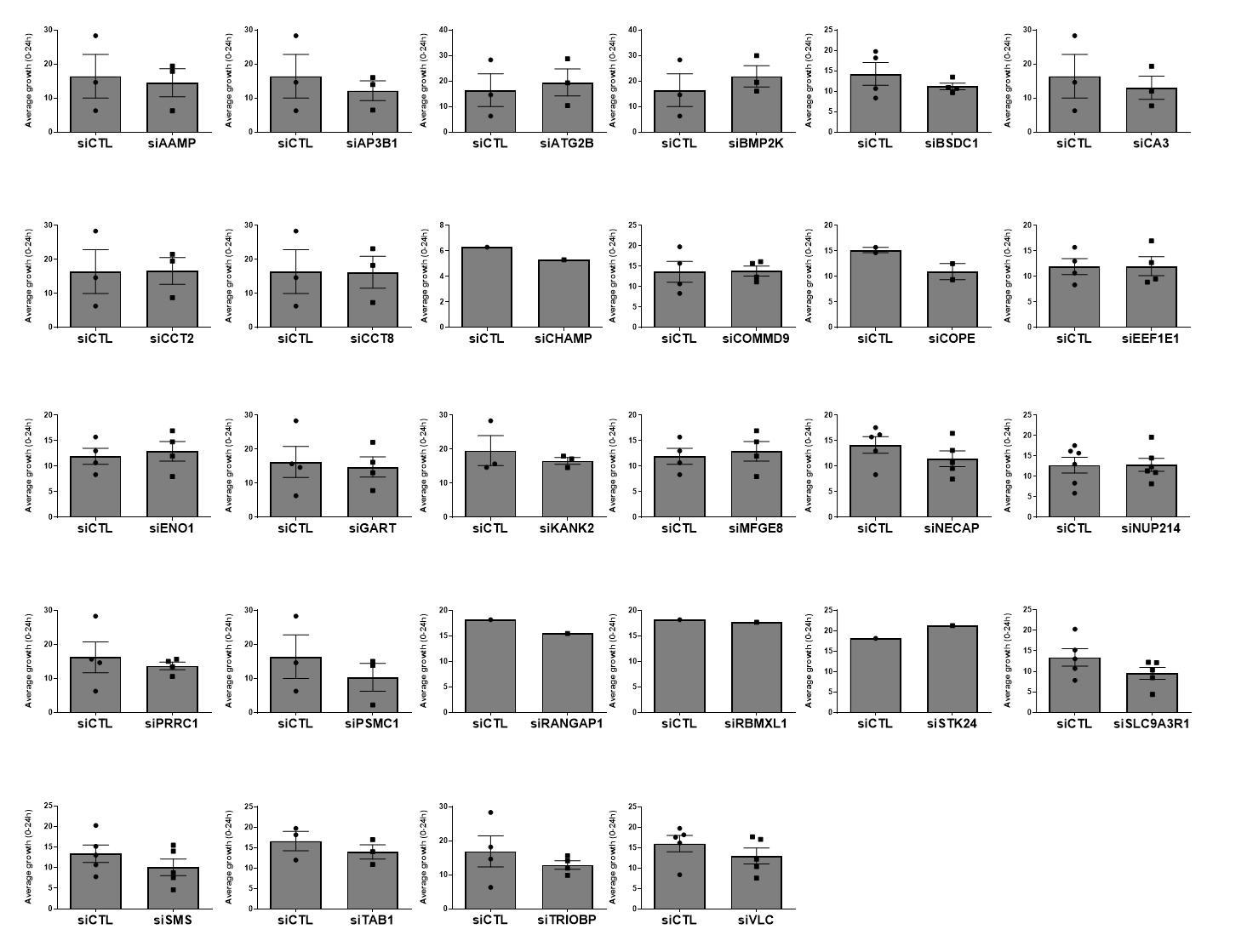

Supplement: Supplementary Figure 3 — Knockdown of twenty-eight genes did not significantly affect RA FLS mobility in the wound healing (scratch) assay (2-6 different cell lines per treatment condition; shown as mean ± SEM). [file Image_3.tif]

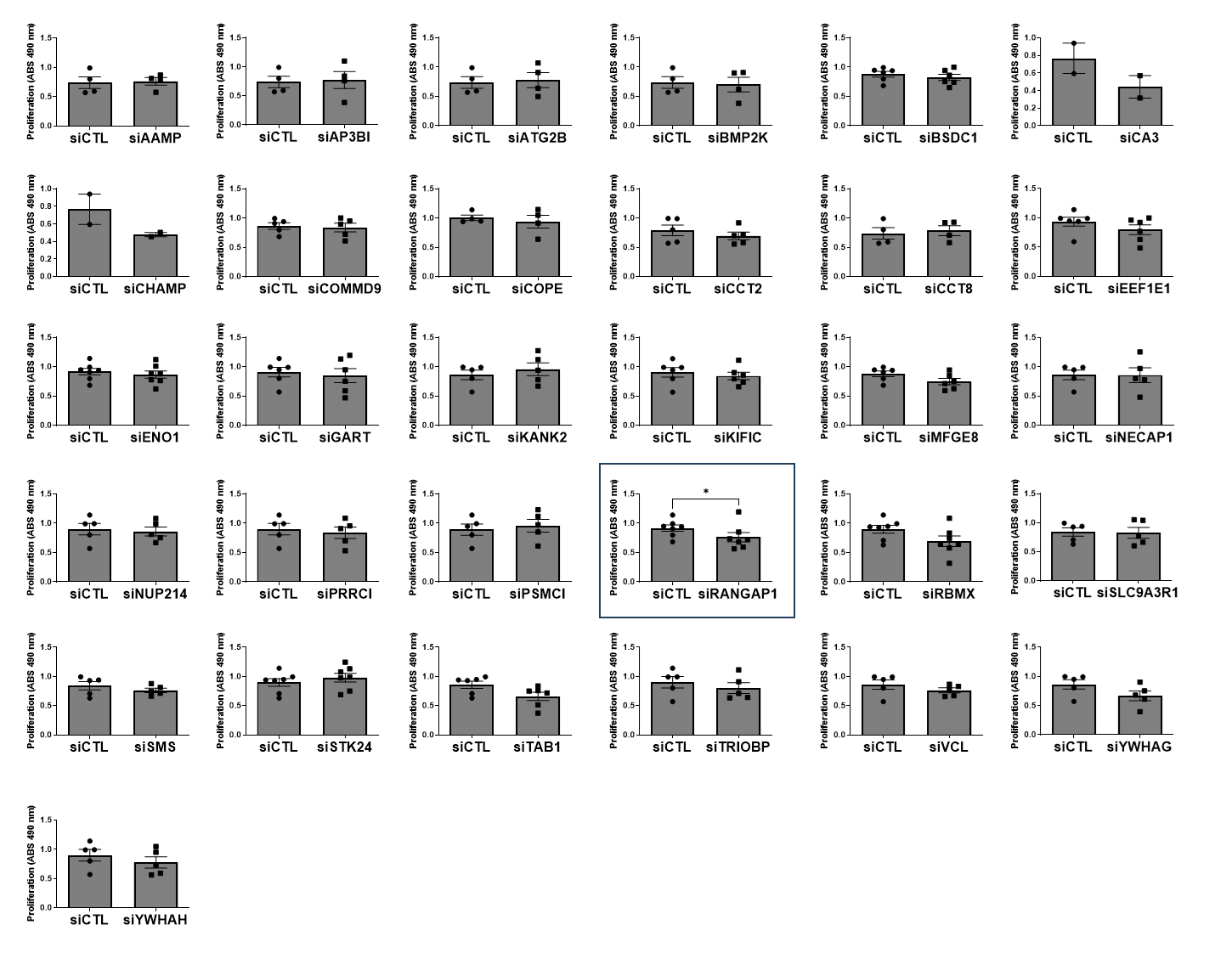

Supplement: Supplementary Figure 4 — Only one (RANGAP1) of the thirty-one genes tested significantly affected RA FLS proliferation. Knockdown of only one gene (marked with a square) significantly affected (reduced) RA FLS proliferation (*P ≤ 0.05), (2-7 different cell lines per treatment condition; shown as mean ± SEM). [file Image_4.tif]

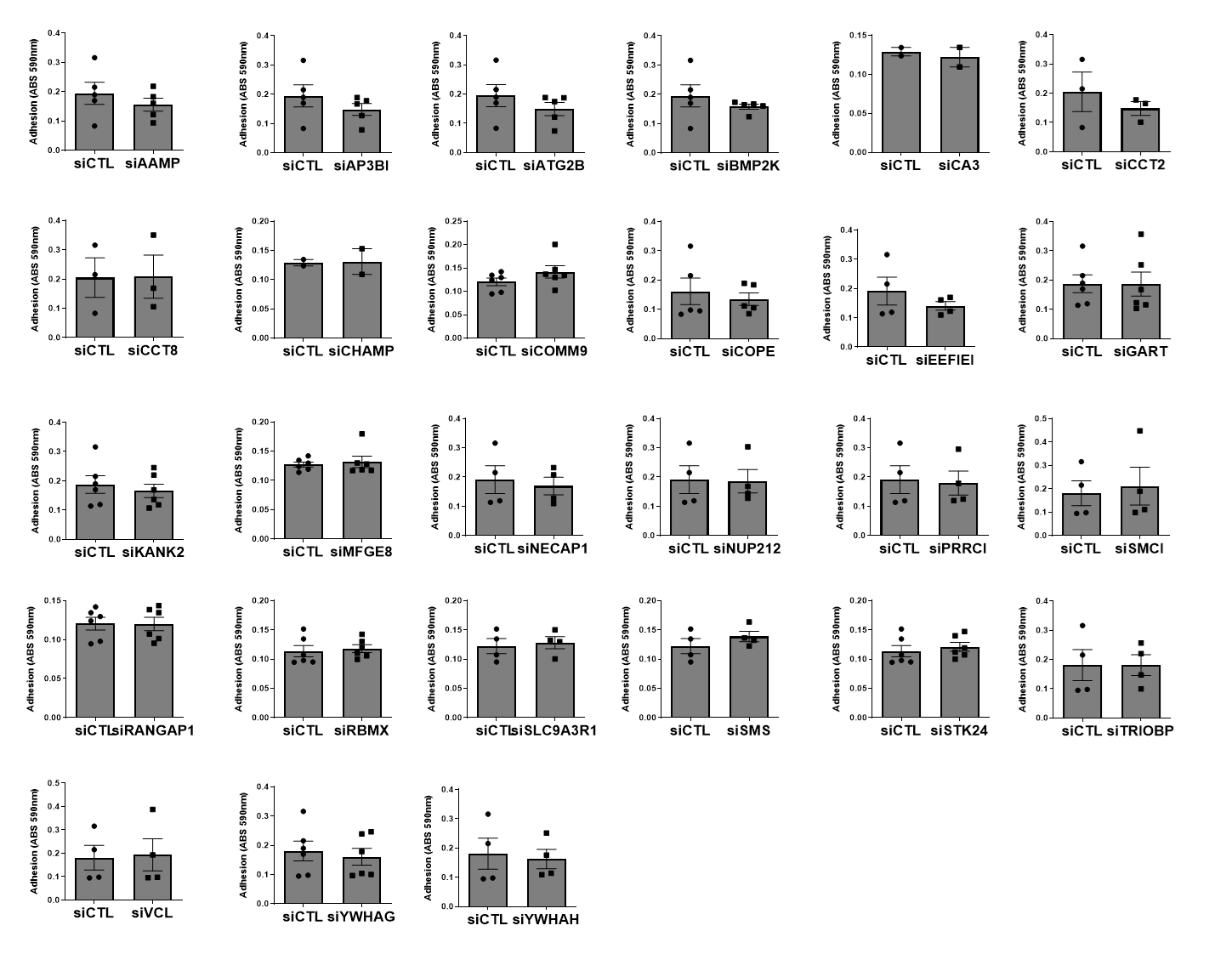

Supplement: Supplementary Figure 5 — Adhesion assay of twenty-seven genes. None of these genes significantly affected RA FLS adhesion (2-6 different cell lines per treatment condition; shown as mean ± SEM). [file Image_5.tif]

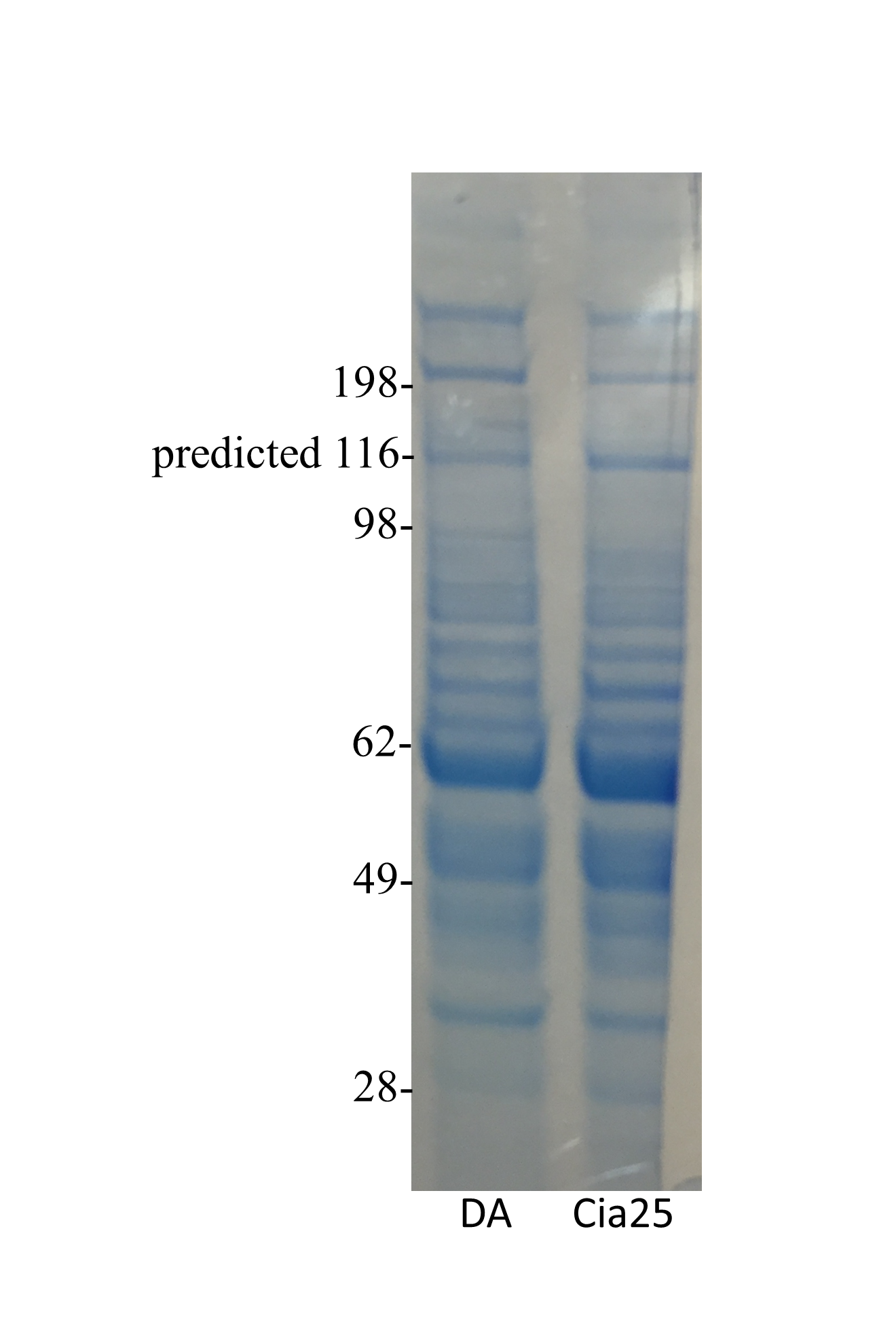

Supplement: Supplementary Figure 6 — Protein from cell extracts from DA and R6 were quantified and identical amounts loaded into a protein gel for electrophoresis (10μl of a 1μg/μl solution) followed by staining with Commassie blue. HIP1 is predicted to migrate into the position 116. [file Image_6.tif]
